# Supplementary material for: Genomic screening of 16 UK native bat species through conservationist networks uncovers coronaviruses with zoonotic potential
Source: Nat Commun. 2023 Jun 27;14:3322. doi: 10.1038/s41467-023-38717-w (PMC10300128; doi:10.1038/s41467-023-38717-w)
Supplement: Supplementary file 1 — Supplementary Information [file 41467_2023_38717_MOESM1_ESM.pdf]

Supplementary Information: Genomic screening of 16 UK native bat species through conservationist networks uncovers coronaviruses with zoonotic potential

Tan et al.

Existing RT-PCR assays underestimate coronavirus prevalence in bats

Given that RT-PCR has conventionally been used to screen for coronaviruses in bats, we sought to determine if the novel coronavirus genomes we recovered from our metatranscriptomes could have been detected using published pan-coronavirus primers. Using BLASTn searches, we aligned the external RT-PCR primers that have been described previously[1–5] against all coronavirus genomes in our custom database and our nine novel genomes. These include primers that have been used widely[1,3,4], and updated primers described in two more recent studies[2,5]. Amongst these primers, the ones designed by Holbrook et al.[5] are an updated version of those by Watanabe et al.[3]. Notably, whether a primer can bind to a particular genomic sequence is difficult to predict *in vitro* since the impact of mismatches on primer binding can depend on various factors such as the position of the mismatch or annealing temperature[6–8]. We therefore assumed that a primer sequence can bind to a coronavirus genome if a primer-genome alignment could be produced by BLASTn, and conversely, that a primer sequence is not likely to bind if no primer-genome alignment could be identified. Under this assumption, the coronavirus diversity that can be 'detected' by each primer set can be estimated by the proportion of coronavirus genomes that could be aligned to a query primer sequence. Since most of these primers contained degenerate bases, we performed the BLASTn analysis on every combination of non-degenerate bases for each primer and retained only the primer-genome alignment with the lowest number of mismatches.

None of the external primer sets, except that by Vijgen et al.[4], were able to detect all nine novel genomes (Supplementary Figure 1a). In fact, three of the external primer sets[1–3] could detect at most one of the novel coronaviruses. We extended this analysis further by analysing the sequence homology of all external primer sets to all genomes in our custom coronavirus database. All external primer sets carried at least one

mismatch or had no detectable homology to at least one coronavirus genome in our database, indicating that none are likely to capture the full existing diversity of coronaviruses (Supplementary Figure 1b). Strikingly, the proportion of coronavirus genomes that could be detected by any external primer set, estimated from the number of detectable primer-genome alignments, ranged from 9.5 to 93.5%. Given that our analysis only includes the external primers, additional mismatches in the internal primer set may exacerbate the poor sensitivity of these RT-PCR assays. Overall, these findings indicate that RT-PCR screens that employ these primers likely underestimate viral prevalence in the systems being studied.

Genome structure analyses indicate the presence of novel genes

We used various bioinformatic tools (see Methods) to determine if these genomes carry any novel genes. No notable novel genes were identified in the sarbecoviruses, which like RhGB01 have a similar genome structure to SARS-CoV-2 and SARS-CoV but are missing ORF8[9] (Supplementary Figure 5). Although RfGB02 has an out of frame deletion that likely results in a truncated ORF7a. Similarly PpiGB02, MdGB02 and MdGB03 had similar genome structures to other bat Pedacoviruses, potentially expressing an additional ORF7 relative to PEDV[10]. The pedacovirus MdGB01 does however contain an additional potential ORF8 at the 3' end of the genome, which is absent in the other UK bat pedacoviruses. This potential ORF8 has an upstream putative transcriptional regulatory sequence (TRS) and would result in expression of a 56 amino acid (a.a.) protein. However, PaGB01 encodes a novel 100 a.a protein that is only 54.9% similar to its closest homologue, the ORF3 accessory protein in MERS-CoV. This putative ORF3-like protein could not be assigned to any InterPro protein families[11], but was predicted to contain a transmembrane and an extracellular domain. PaGB01 also encodes a 218 a.a. protein at 73.3% identity to the MERS-CoV ORF5 protein. Finally PaGB01 also encodes an ORF predicted to express an 83 a.a. protein, partially overlapping (in the +1 reading frame) with its N gene at the 3' end of its genome. Consistent with coronavirus gene naming conventions, this would be named ORF8c. The divergence of these novel proteins from MERS-CoV are largely in line with that between the accessory proteins in MERS-CoV and other bat-borne MERS-CoV-related species, btCoV-HKU4 and btCoV-HKU5[12]. This indicates that the novel proteins may possess functions similar to the MERS-CoV accessory proteins.

Accessory proteins are non-essential for coronavirus replication *in vitro*, but are thought to play key roles in host-virus interactions. For example, ORF3 and ORF5 proteins in MERS-CoV have been shown to induce apoptosis[13] and also to antagonise interferon responses[14], which are a key aspect of the innate immune response to viruses in humans. The accessory genes of coronaviruses are highly variable in number and function across the family *Coronaviridae*. However, MERS-CoV and its close bat-borne relatives, btCoV-HKU4 and btCoV-HKU5, share a similar number of accessory genes with similar functions, despite low protein sequence similarities between the accessory proteins from these species[12,15]. In light of this, further characterisation of the novel proteins identified in PaGB01 may reveal fundamental insights on the evolution of viral pathogenicity. For example, if the accessory genes of PaGB01 match the function of the MERS-CoV equivalent proteins in interacting with human cellular signalling pathways, that could suggest that immunoregulation is a conserved function amongst MERS-CoV-related coronaviruses and may help explain how MERS-CoV is able to cause human disease. Conversely, a lack of shared activity may indicate that these functions are unique to MERS-CoV and its closest relatives and are not universally found in other sister lineages, perhaps explaining why there is no evidence of other MERS-related virus infections in humans to date.

High prevalence of recombination amongst sarbecoviruses

Given that further adaptations are necessary for the zoonotic emergence of RhGB01-like viruses, we asked if genetic recombination may speed up this process. Recombination in viruses allows the genetic transfer of large sections of the genome in a single event, helping them sample the genomic sequence space at a more rapid pace when compared to the accumulation of point mutations alone[16]. In fact several regions in the spike protein of coronaviruses that influence host range have been suggested to have been acquired through recombination[17], which implies that recombination may be an important driver for zoonotic emergence. As such, we performed recombination analyses for sarbecoviruses, including our novel sequences, using the recombination detection program (RDP)[18]. This tool comprises a suite of algorithms for recombination detection and has been used previously for sarbecoviruses[19,20]. We searched for recombination amongst 218 representative

sarbecovirus genomes using all nine algorithms implemented within RDP4 (RDP[21], GENECONV[22], BOOTSCAN[23], MaxChi[24], Chimaera[25], SisScan[26], PhylPro[27], LARD[28] and 3SEQ[29]), retaining predicted breakpoints supported by at least six of these methods. Using this approach, we detected 202 putative recombination events amongst the sarbecoviruses considered, suggesting a high prevalence of recombination within the subgenus. Additionally, we detect an overrepresentation of recombination signals near the N-terminal half of the spike protein (Supplementary Figure 11a), which also contains the receptor binding domain that is the primary determinant of host receptor usage. We also identified six recombination events within the RhGB01-like viruses supported by 2-6 detection algorithms (Supplementary Figure 11b), demonstrating the potential for recombination involving the novel UK sarbecoviruses. Overall, these results support frequent events of recombination in sarbecoviruses, which may increase the likelihood of novel sarbecoviruses, some which may be zoonotic, emerging in *Rhinolophus* bats in the UK.

Supplementary Figures

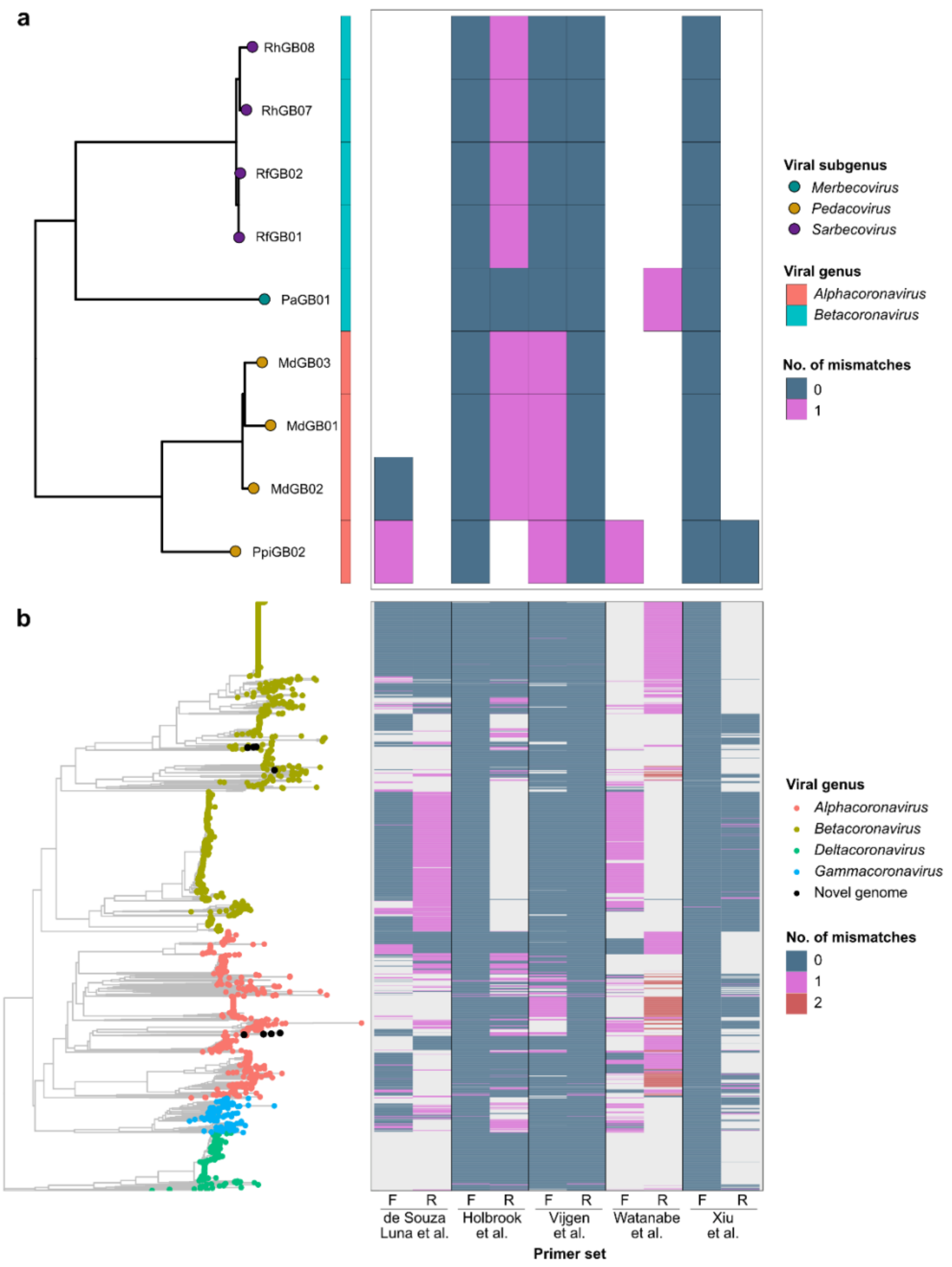

Supplementary Figure 1. RT-PCR assays underestimate coronavirus prevalence. Heatmap summarising the number of mismatches of the forward (F) and reverse (R) degenerate primers described in previous studies to (a) novel genomes, and (b) to the nine novel and 2118 genomes in our custom coronavirus database. Both heatmaps are matched to the tips of the alignment-free trees generated from the genomes analysed, which are similar to that shown in Fig. 1a but represented as a linear phylogram. Heatmap cells coloured white or gray indicate no detectable homology between a degenerate primer and a genome by BLASTn.

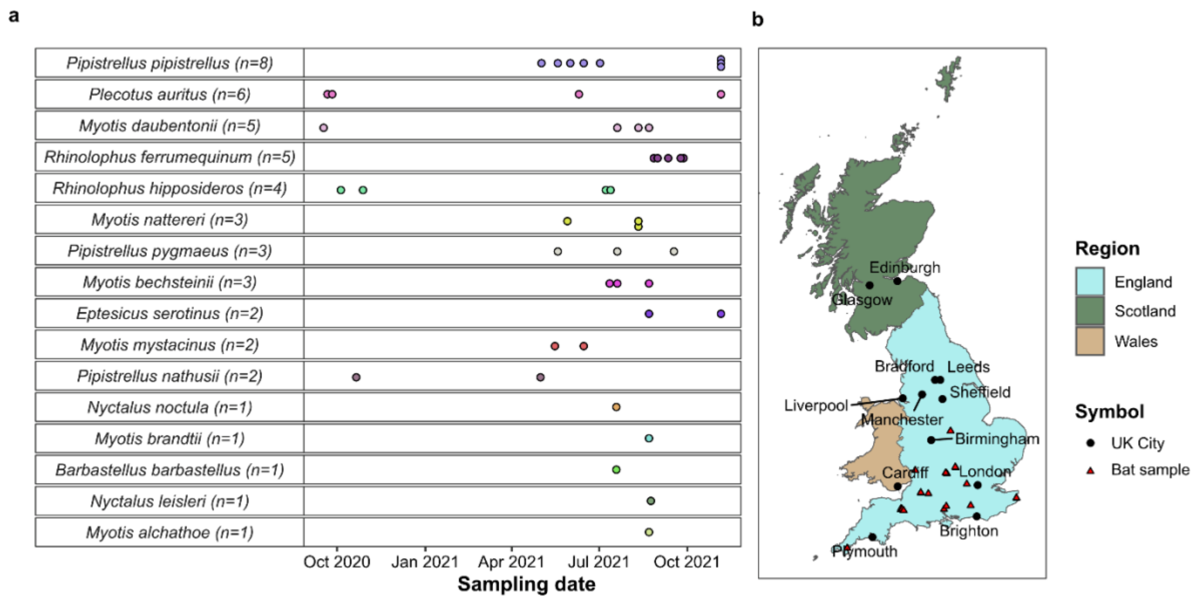

Supplementary Figure 2. Collection of faecal samples from 16 UK bat species through extensive network of bat rehabilitators. (a) Temporal distribution of samples collected with the number of samples per host species annotated. (b) Geographical distribution of samples collected relative to the major cities in the UK.

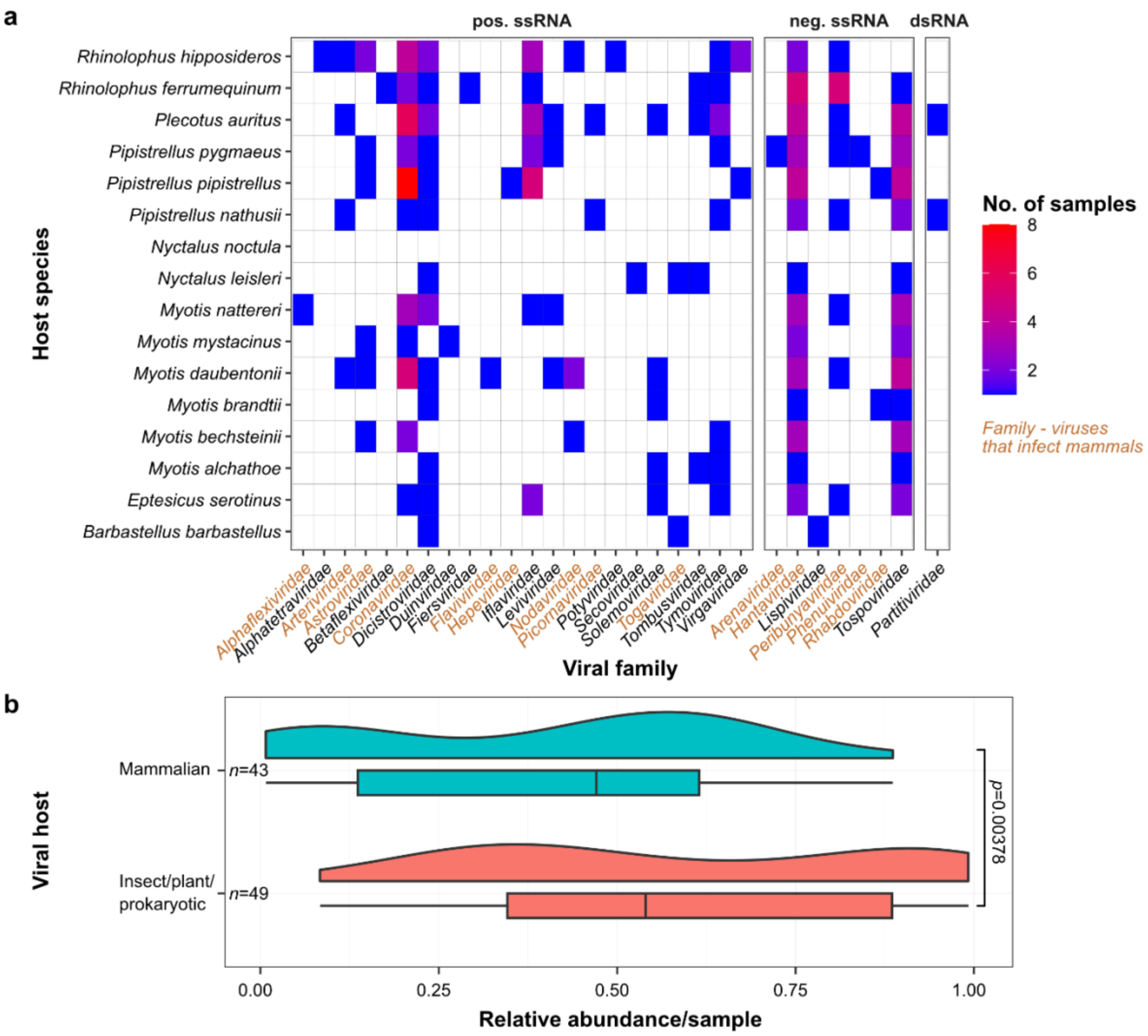

Supplementary Figure 3. Analysis of the UK bat faecal virome. (a) Heatmap summarizing the number of samples per UK bat species where a particular viral family was present, based on Kraken2 taxonomic assignment of reads. Viral families that are known to infect mammals are highlighted in brown. (b) The total relative abundance of mammalian or non-mammalian viral species in each sample. Data are visualized with both Gaussian kernel probability density and box-and-whisker plots (centre line, median; box limits, upper and lower quartiles; whiskers, 1.5x interquartile range). A two-sided Mann-Whitney U test was used to test if the two distributions differed.

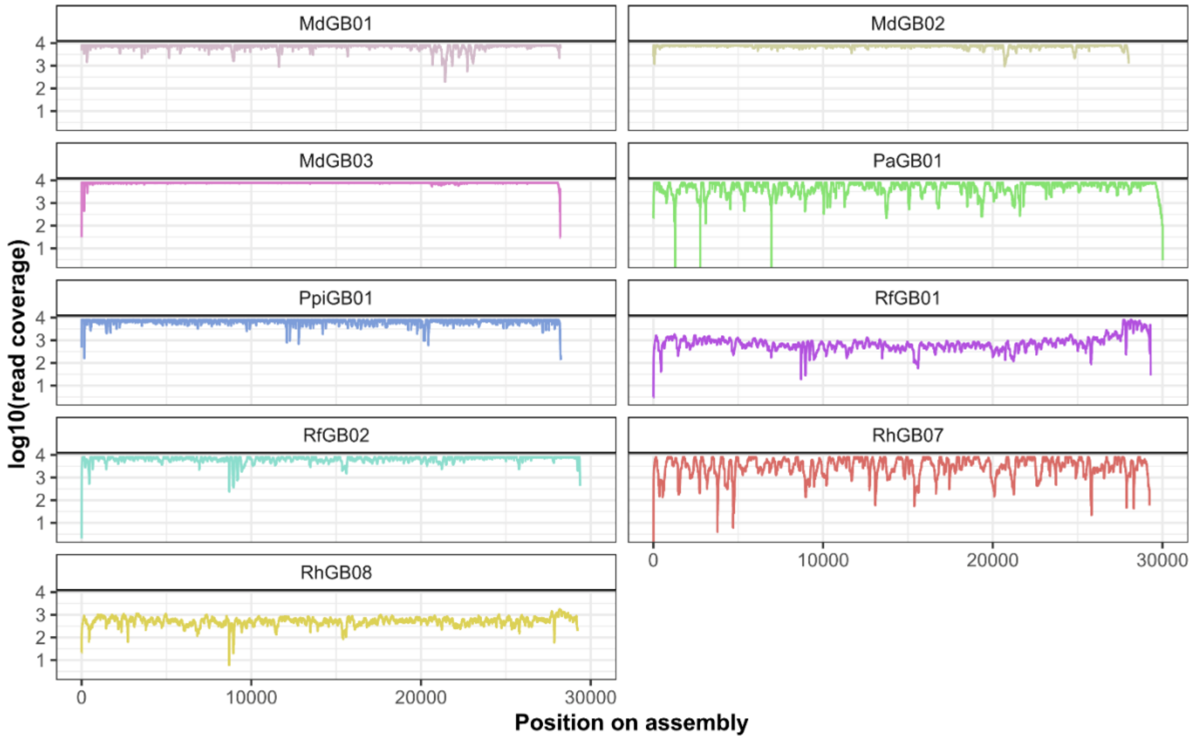

Supplementary Figure 4. Even read coverage across all complete genomes recovered from UK bats. Sequencing reads were mapped back to the final genomes using Bowtie2 and per-position read coverage was calculated using Samtools.

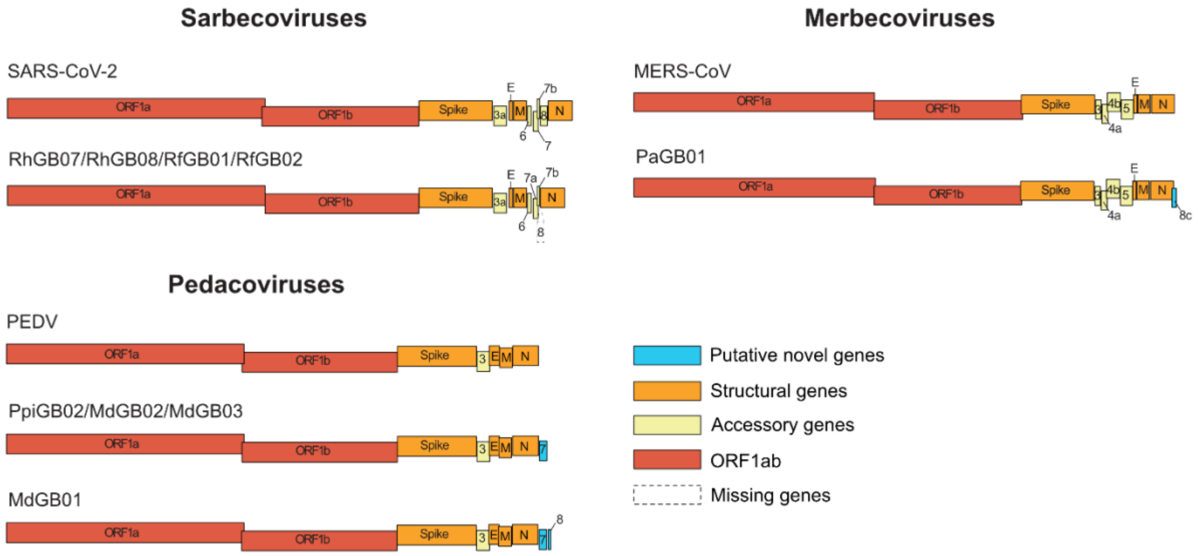

Supplementary Figure 5. Genome schematics of the novel UK bat coronaviruses. To-scale layouts of ORFs within the novel bat coronaviruses from this study compared to prototypic genomes from the same subgenera. ORF1ab polyproteins are shown in red, structural proteins in orange, accessory proteins in yellow, and putative novel ORFs in blue. Missing ORFs relative to the prototypes shown by dotted lines. Standard coronavirus gene nomenclature was used throughout. This figure was made using Adobe Illustrator v27.1.1 and Geneious v11.1.5 (https://www.geneious.com).

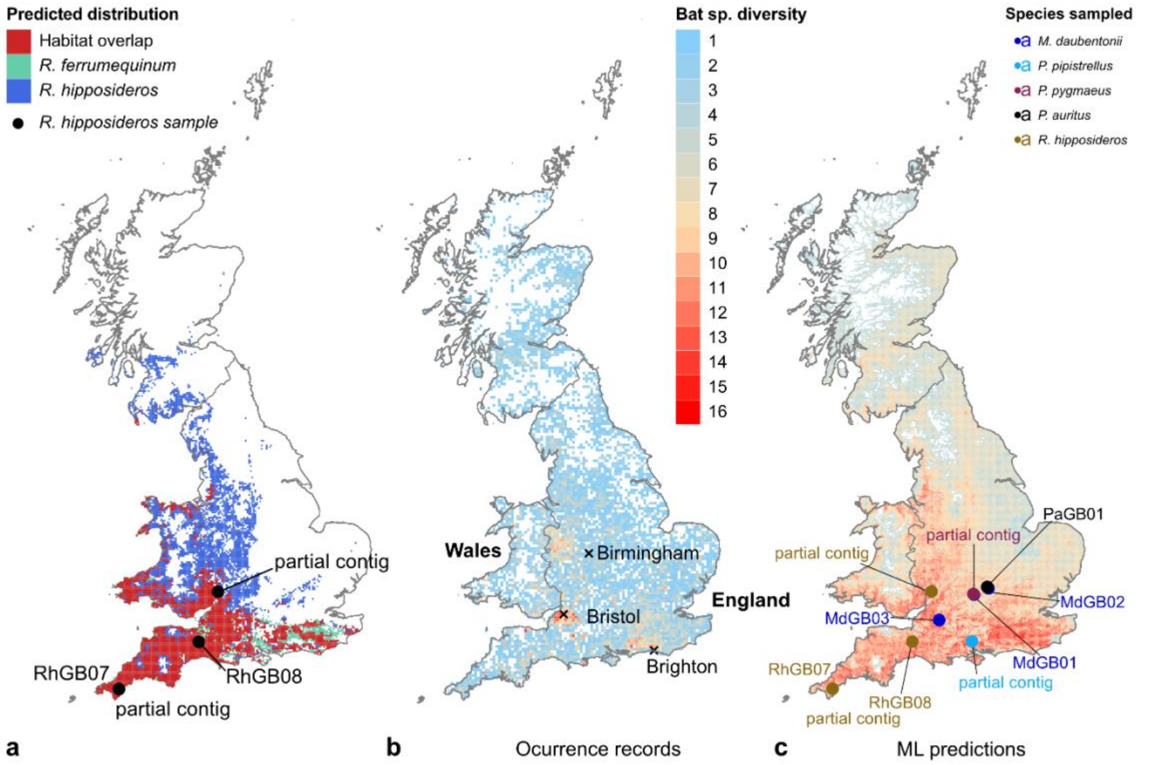

Supplementary Figure 6. Species distribution maps of UK bats. (a) Predicted distributions of *R. ferrumequinum* and *R. hipposideros* species in the UK. (b) Species diversity (i.e., number of species) found within a 5x5 km square grid computed based on occurrence records dating from 2000-present. (c) Predicted species diversity all 17 UK breeding bat species found within a 1x1 km square grid. All predicted distributions were generated by our ensemble machine learning model. Species were deemed to be present if the predicted probability score (i.e., habitat suitability) generated for any square grid exceeds 0.8. *Rhinolophus* samples and all UK bat samples where coronavirus genomes or partial contigs were recovered, and whose exact geographical coordinates were available are annotated in (a) and (c), respectively.

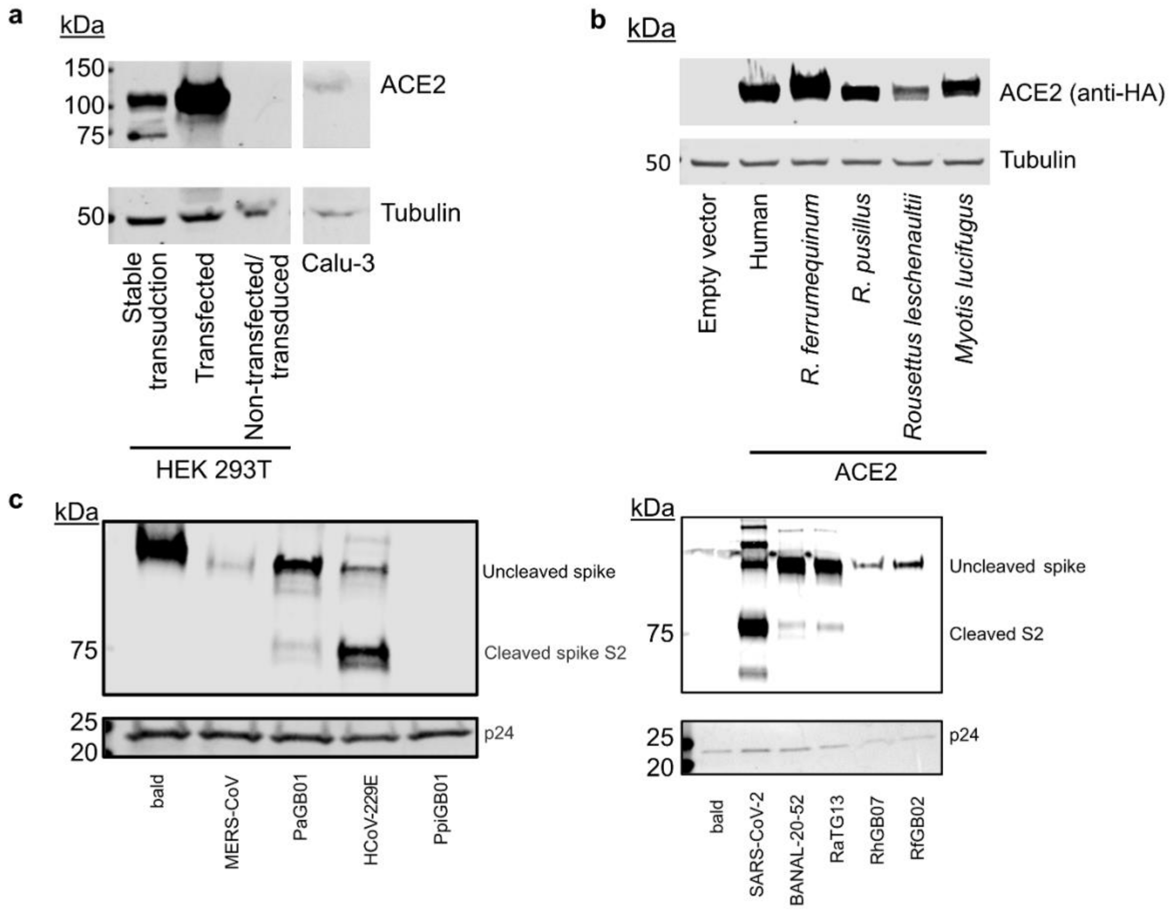

Supplementary Figure 7. Western blot analyses of spike pseudoviruses and cell receptor expression. (a) Western blot showing relative ACE2 expressions of stably transduced, transfected or non-transfected/transduced HEK293T. (b) Western blot analysis of HEK293T cells transfected with different ACE2 constructs. All ACE2 proteins tagged with C-terminal HA tag. Equal loading shown by probing with anti-tubulin antibody. (c) Western blot analysis of concentrated pseudovirus expressing different sarbecovirus, merbecovirus and pedacovirus spike proteins. Sarbecovirus spike expression (upper panel) determined by a pan-sarbecovirus anti-S2 antibody. Pedacovirus and merbecovirus spike expression determined by incorporatation of C-terminally Myc-tagged spike (lower panel). The upper band corresponds to uncleaved, full length spike, the lower band to the cleaved S2 fragment. Loading shown by p24 lentiviral capsid protein. All western blots shown are representative repeats of n=3 independent experiments performed.

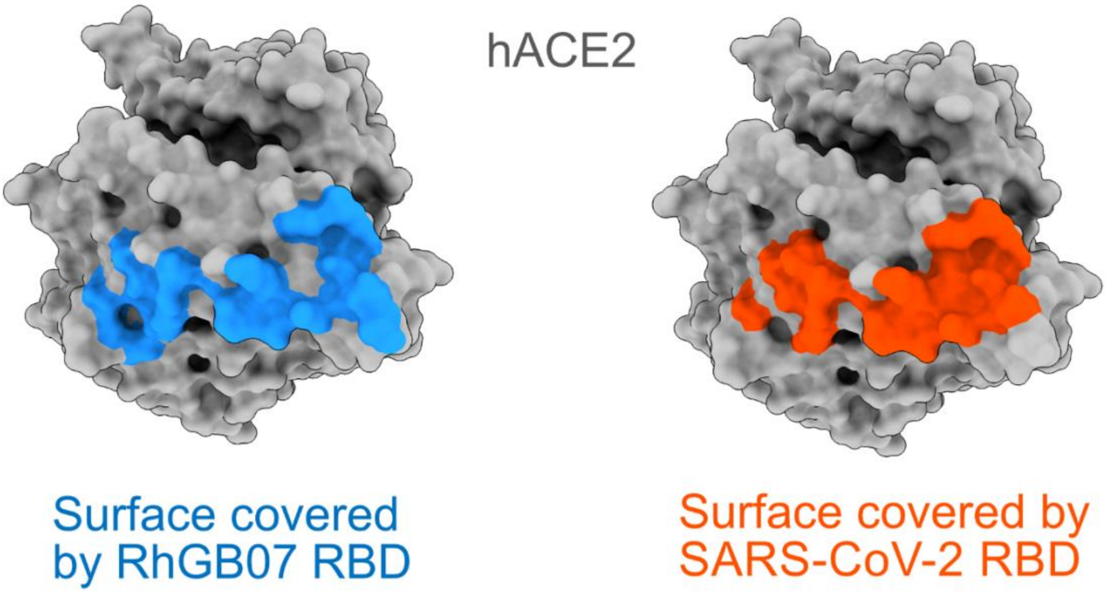

Supplementary Figure 8. Protein surfaces of hACE2 in contact with RhGB07 or SARS-CoV-2 receptor-binding domain (RBD). The structure of hACE2 is shown in grey and the surface in contact with the RBDs of RhGB07 (blue) and SARS-CoV-2 (orange) are highlighted. We computed the surface are of hACE2 in contact with either RhGB07 or SARS-CoV-2 RBD using the *buriedarea* command in *ChimeraX.*

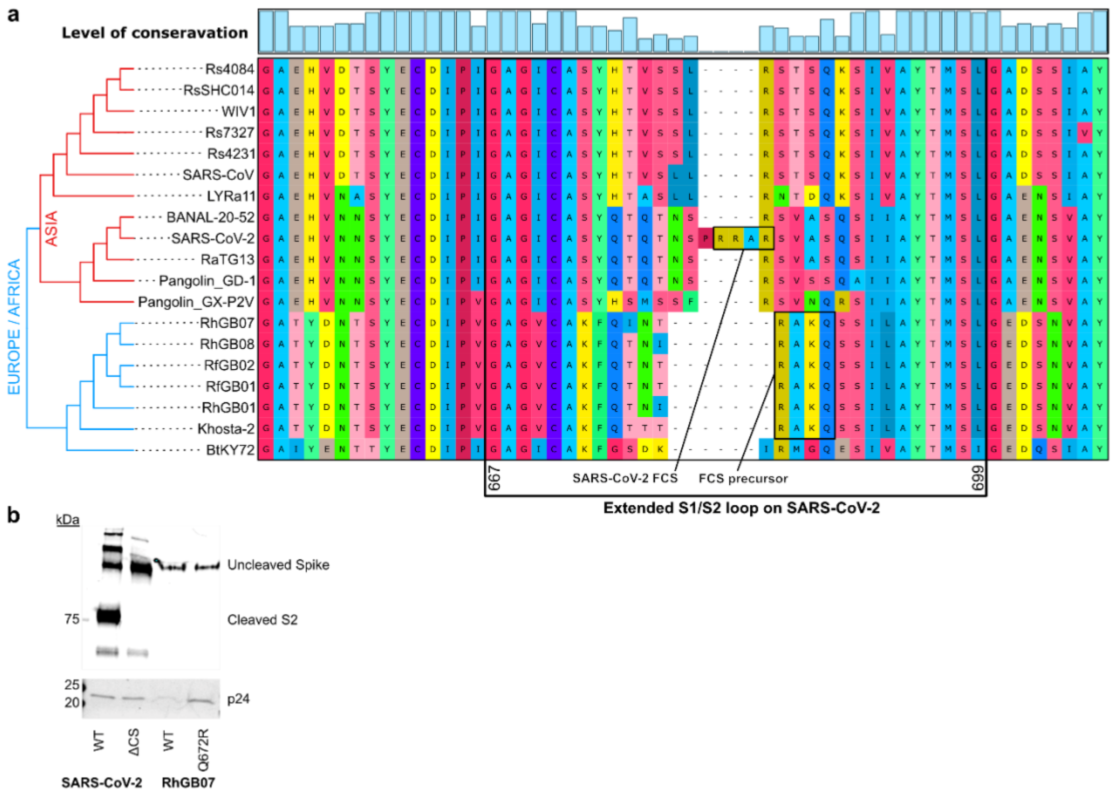

Supplementary Figure 9. European sarbecoviruses posses an RAKQ motif resembling a furin cleavage site. (a) Sequence alignment of sarbecovirus spike genes at the region surrounding the SARS-CoV-2 furin cleavage site (FCS) and R-A-K-Q furin cleavage site precursor in UK sarbecoviruses. Sequence alignment was visualized using UGENE v42.0. The alignment region comprising SARS-CoV-2 spike residue positions 667-699 is indicated by a black rectangle and corresponds to the extended S1/S2 loop containing the R-R-A-R FCS present in SARS-CoV-2. Barchart showing the proportion of genomes with residues identical to SARS-CoV-2 at each position (top). Maximum-likelihood tree identical to that shown in Fig. 3c (left) showing the genetic relatedness of Asian, European and African sarbecoviruses. (b) Western blot of RhGB07 spike with or without the Q672R mutation (generating an RAKR motif). SARS-CoV-2 spike with or without the 678-NSPRRARS-687 deletion were used as negative and positive controls, respectively.

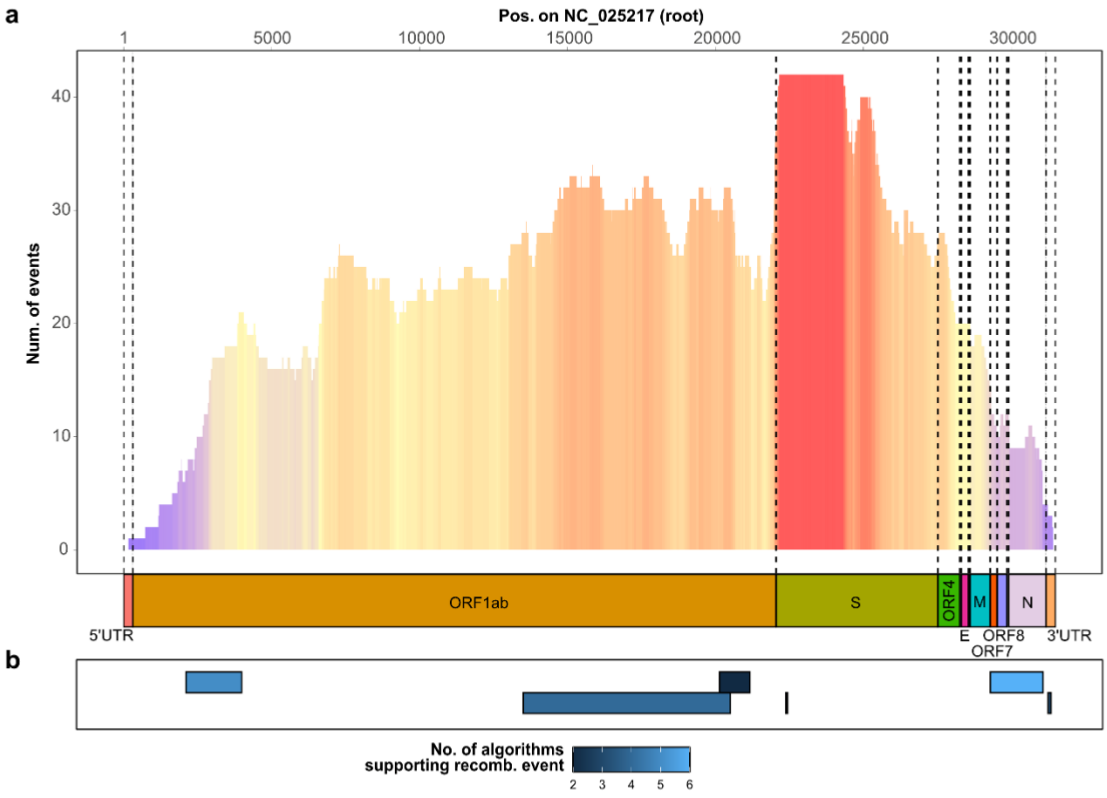

Supplementary Figure 10. High prevalence of recombination amongst sarbecoviruses. (a) Distribution of recombination events detected by at least six of the nine recombination detection algorithms in RDP4. This analysis was performed on an alignment of 218 representative sarbecoviruses, including RhGB01 and our four novel sarbecoviruses (RhGB07, RhGB08, RfGB01, RfGB02), using NC_025217 as the reference. (b) All recombination events involving RhGB01-like viruses either as donor or recipients. Recombination events were supported by 2-6 detection algorithms.

(ATTACHED AS SEPARATE PDF)

Supplementary Figure 11. Species distribution modelling for the 17 UK breeding bat species. (Left) Performance of individual machine-learning algorithms in predicting species distributions. (Right) Maps of individual species distributions. Predicted probability scores indicate the predicted habitat suitability for each 1x1km square grid, which ranges from 0 (unsuitable habitat) to 1 (suitable habitat). The number of occurrence records for each bat species used to train the models, and the geographical locations of bat samples collected in this study are indicated.

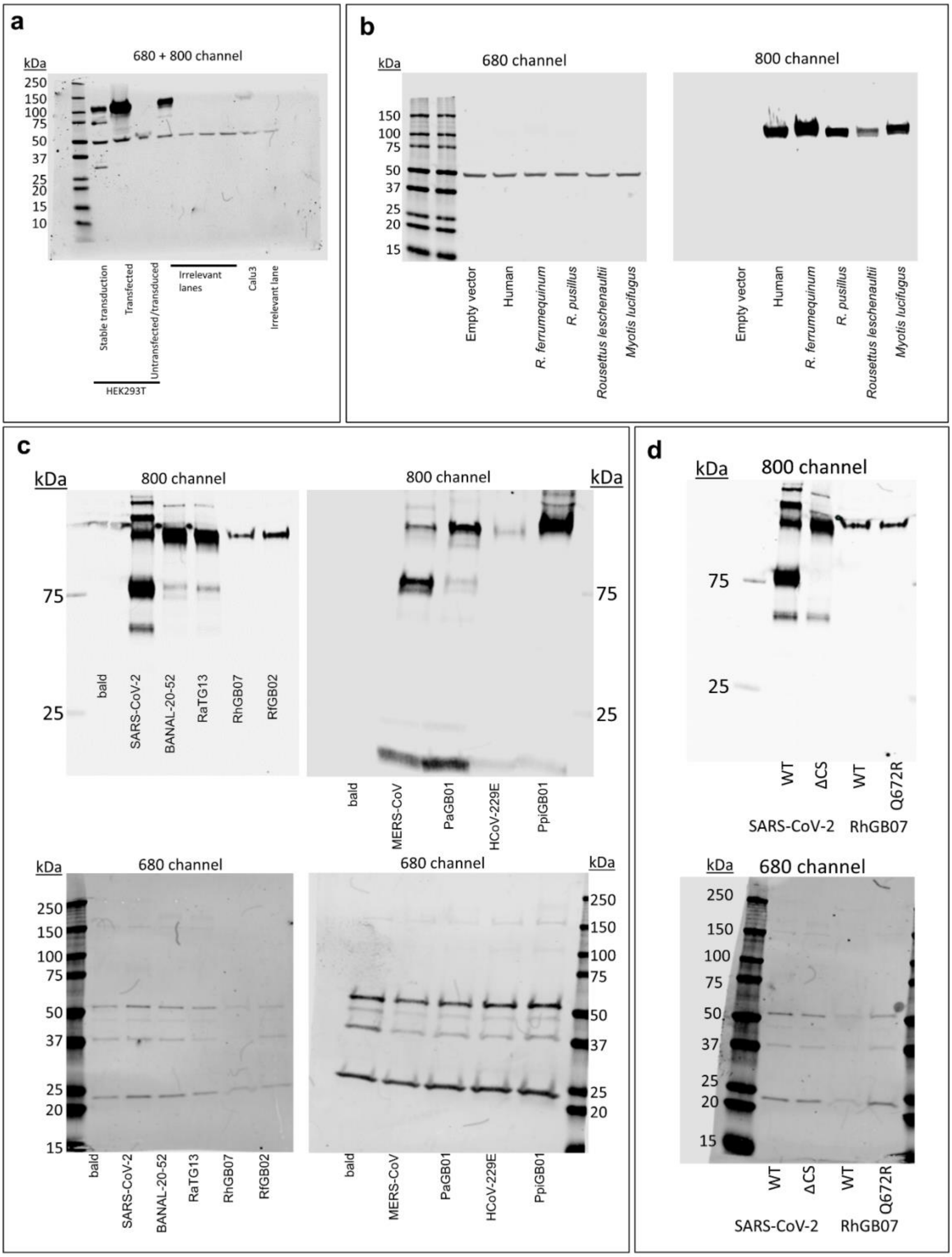

Supplementary Figure 12. Raw uncropped images of western blots. Panels (a), (b), (c) and (d) correspond to the images shown in Supplementary Fig. 7a, 7b, 7c and 9b, respectively.

References

1. De Souza Luna, L. K. *et al.* Generic Detection of Coronaviruses and Differentiation at the Prototype Strain Level by Reverse Transcription-PCR and Nonfluorescent Low-Density Microarray. *J. Clin. Microbiol.* **45**, 1049 (2007).
2. Xiu, L. *et al.* A RT-PCR assay for the detection of coronaviruses from four genera. *J. Clin. Virol.* **128**, 104391 (2020).
3. Watanabe, S. *et al.* Bat coronaviruses and experimental infection of bats, the Philippines. *Emerg. Infect. Dis.* **16**, 1217 (2010).
4. Vijgen, L., Moës, E., Keyaerts, E., Li, S. & Ranst, M. V. A pancoronavirus RT-PCR assay for detection of all known coronaviruses. in *SARS-and Other Coronaviruses* 3–12 (Springer, 2008).
5. Holbrook, M. G. *et al.* Updated and validated pan-coronavirus PCR assay to detect all coronavirus genera. *Viruses* **13**, 599 (2021).
6. Ye, J. *et al.* Primer-BLAST: A tool to design target-specific primers for polymerase chain reaction. *BMC Bioinformatics* **13**, 134 (2012).
7. Waterfall, C. M., Eisenthal, R. & Cobb, B. D. Kinetic characterisation of primer mismatches in allele-specific PCR: a quantitative assessment. *Biochem. Biophys. Res. Commun.* **299**, 715–722 (2002).
8. Whiley, D. M. & Sloots, T. P. Sequence variation in primer targets affects the accuracy of viral quantitative PCR. *J. Clin. Virol.* **34**, 104–107 (2005).
9. Crook, J. M. *et al.* Metagenomic identification of a new sarbecovirus from horseshoe bats in Europe. *Sci. Rep.* **11**, 1–9 (2021).
10. Lo, V. T., Yoon, S. W., Choi, Y. G., Jeong, D. G. & Kim, H. K. Genomic Comparisons of Alphacoronaviruses and Betacoronaviruses from Korean Bats. *Viruses* **14**, 1389 (2022).

11. Blum, M. *et al.* The InterPro protein families and domains database: 20 years on. *Nucleic Acids Res.* **49**, D344–D354 (2021).
12. Matthews, K. L., Coleman, C. M., van der Meer, Y., Snijder, E. J. & Frieman, M. B. The ORF4b-encoded accessory proteins of Middle East respiratory syndrome coronavirus and two related bat coronaviruses localize to the nucleus and inhibit innate immune signalling. *J. Gen. Virol.* **95**, 874 (2014).
13. Zhou, Y. *et al.* Host E3 ligase HUWE1 attenuates the proapoptotic activity of the MERS-CoV accessory protein ORF3 by promoting its ubiquitin-dependent degradation. *J. Biol. Chem.* **298**, (2022).
14. Yang, Y. *et al.* The structural and accessory proteins M, ORF 4a, ORF 4b, and ORF 5 of Middle East respiratory syndrome coronavirus (MERS-CoV) are potent interferon antagonists. *Protein Cell* **4**, 951–961 (2013).
15. Woo, P. C., Lau, S. K., Li, K. S., Tsang, A. K. & Yuen, K.-Y. Genetic relatedness of the novel human group C betacoronavirus to Tylonycteris bat coronavirus HKU4 and Pipistrellus bat coronavirus HKU5. *Emerg. Microbes Infect.* **1**, 1–5 (2012).
16. Simon-Loriere, E. & Holmes, E. C. Why do RNA viruses recombine? *Nat. Rev. Microbiol.* **9**, 617–626 (2011).
17. Graham, R. L. & Baric, R. S. Recombination, reservoirs, and the modular spike: mechanisms of coronavirus cross-species transmission. *J. Virol.* **84**, 3134–3146 (2010).
18. Martin, D. P., Murrell, B., Golden, M., Khoosal, A. & Muhire, B. RDP4: Detection and analysis of recombination patterns in virus genomes. *Virus Evol.* **1**, (2015).
19. Boni, M. F. *et al.* Evolutionary origins of the SARS-CoV-2 sarbecovirus lineage responsible for the COVID-19 pandemic. *Nat. Microbiol.* **5**, 1408–1417 (2020).

20. Lytras, S. *et al.* Exploring the natural origins of SARS-CoV-2 in the light of recombination. *Genome Biol. Evol.* **14**, evac018 (2022).
21. Martin, D. & Rybicki, E. RDP: detection of recombination amongst aligned sequences. *Bioinformatics* **16**, 562–563 (2000).
22. Padidam, M., Sawyer, S. & Fauquet, C. M. Possible emergence of new geminiviruses by frequent recombination. *Virology* **265**, 218–225 (1999).
23. Salminen, M. O., Carr, J. K., Burke, D. S. & McCUTCHAN, F. E. Identification of breakpoints in intergenotypic recombinants of HIV type 1 by bootscanning. *AIDS Res. Hum. Retroviruses* **11**, 1423–1425 (1995).
24. Smith, J. M. Analyzing the mosaic structure of genes. *J. Mol. Evol.* **34**, 126–129 (1992).
25. Posada, D. & Crandall, K. A. Evaluation of methods for detecting recombination from DNA sequences: computer simulations. *Proc. Natl. Acad. Sci.* **98**, 13757–13762 (2001).
26. Gibbs, M. J., Armstrong, J. S. & Gibbs, A. J. Sister-scanning: a Monte Carlo procedure for assessing signals in recombinant sequences. *Bioinformatics* **16**, 573–582 (2000).
27. Weiller, G. F. Phylogenetic profiles: a graphical method for detecting genetic recombinations in homologous sequences. *Mol. Biol. Evol.* **15**, 326–335 (1998).
28. Holmes, E. C., Worobey, M. & Rambaut, A. Phylogenetic evidence for recombination in dengue virus. *Mol. Biol. Evol.* **16**, 405–409 (1999).
29. Boni, M. F., Posada, D. & Feldman, M. W. An exact nonparametric method for inferring mosaic structure in sequence triplets. *Genetics* **176**, 1035–1047 (2007).
