# Supplementary material for: Genomic screening of 16 UK native bat species through conservationist networks uncovers coronaviruses with zoonotic potential
Source: Nat Commun. 2023 Jun 27;14:3322. doi: 10.1038/s41467-023-38717-w (PMC10300128; doi:10.1038/s41467-023-38717-w)
Supplement: Supplementary file 3 — Description of Additional Supplementary Files [file 41467_2023_38717_MOESM3_ESM.pdf]

Description of Additional Supplementary Files

File Name: Supplementary Data 1
Description: Summary of a selection of coronavirus surveillance studies in bats.

File Name: Supplementary Data 2
Description: Amino acid changes of RfGB02 from RhGB08.

File Name: Supplementary Data 3
Description: Metadata of all samples sequenced by deep RNA sequencing.

File Name: Supplementary Data 4
Description: Metadata of all 2118 coronavirus genomes from NCBI and GISAID used in this study.

File Name: Supplementary Data 5
Description: List of NBN Atlas datasets used in this study.

File Name: Supplementary Data 6
Description: GISAID acknowledgement table for GISAID genomes used in this study.
