# Supplementary material for: Genomic screening of 16 UK native bat species through conservationist networks uncovers coronaviruses with zoonotic potential
Source: Nat Commun. 2023 Jun 27;14:3322. doi: 10.1038/s41467-023-38717-w (PMC10300128; doi:10.1038/s41467-023-38717-w)
Supplement: Supplementary file 6 — Reporting Summary [file 41467_2023_38717_MOESM6_ESM.pdf]

nature portfolio

Corresponding author(s): Vincent Savolainen

Last updated by author(s): Apr 26, 2023

Reporting Summary

Nature Portfolio wishes to improve the reproducibility of the work that we publish. This form provides structure for consistency and transparency in reporting. For further information on Nature Portfolio policies, see our Editorial Policies and the Editorial Policy Checklist.

Statistics

For all statistical analyses, confirm that the following items are present in the figure legend, table legend, main text, or Methods section.

n/a	Confirmed	
☐	☒	The exact sample size (*n*) for each experimental group/condition, given as a discrete number and unit of measurement
☐	☒	A statement on whether measurements were taken from distinct samples or whether the same sample was measured repeatedly
☐	☒	The statistical test(s) used AND whether they are one- or two-sided<br>*Only common tests should be described solely by name; describe more complex techniques in the Methods section.*
☒	☐	A description of all covariates tested
☐	☒	A description of any assumptions or corrections, such as tests of normality and adjustment for multiple comparisons
☐	☒	A full description of the statistical parameters including central tendency (e.g. means) or other basic estimates (e.g. regression coefficient) AND variation (e.g. standard deviation) or associated estimates of uncertainty (e.g. confidence intervals)
☐	☒	For null hypothesis testing, the test statistic (e.g. *F*, *t*, *r*) with confidence intervals, effect sizes, degrees of freedom and *P* value noted<br>*Give P values as exact values whenever suitable.*
☒	☐	For Bayesian analysis, information on the choice of priors and Markov chain Monte Carlo settings
☒	☐	For hierarchical and complex designs, identification of the appropriate level for tests and full reporting of outcomes
☒	☐	Estimates of effect sizes (e.g. Cohen's *d*, Pearson's *r*), indicating how they were calculated

Our web collection on statistics for biologists contains articles on many of the points above.

Software and code

Policy information about availability of computer code

Data collection

Bat occurrence records data were gathered from the online databases NBN Atlas (https://nbnatlas.org/) and GBIF (www.gbif.org).
Existing genomes were downloaded from GISAID and NCBI Virus.

Data analysis

Geneious v11.1.5
bbduk.sh v39.01
coronaSPAdes v3.15.4
Prokka v1.14.6
sdm v1.1.8
Mash v2.3
Ape v5.6.2
Augur v14.0.0
MAFFT v7.490
IQTree v2.1.4-beta
FigTree v1.4.4
ggtree v3.2.1
RDP v4.101
Biostrings v2.62.0
UGENE v42.0
ColabFold v1.3.0
ChimeraX v1.5
Bowtie2 v2.4.5
Geneious v11.1.5
PSI-BLAST web server (no version number; https://blast.ncbi.nlm.nih.gov/)
InterProScan web server (no version number; https://www.ebi.ac.uk/interpro/search/sequence/)

All custom code used to perform the analyses reported here are hosted on GitHub (https://github.com/cednotsed/bat-CoVs.git).

For manuscripts utilizing custom algorithms or software that are central to the research but not yet described in published literature, software must be made available to editors and reviewers. We strongly encourage code deposition in a community repository (e.g. GitHub). See the Nature Portfolio guidelines for submitting code & software for further information.

Data

Policy information about availability of data

All manuscripts must include a data availability statement. This statement should provide the following information, where applicable:

- Accession codes, unique identifiers, or web links for publicly available datasets
- A description of any restrictions on data availability
- For clinical datasets or third party data, please ensure that the statement adheres to our policy

All novel genomes are available in NCBI GenBank under the accessions OQ401247-OQ401251, and OQ401253-OQ401255 (BioProject accession PRJNA929706). The raw sequencing reads generated and analysed in this study have also been uploaded to the SRA under the accessions SRX19257406- SRX19257414. All GenBank and GISAID accessions for the sequences in the coronavirus database are provided in Supplementary Data 4. Other sequences used are as follows: MHV, AY700211.1; BANAL-236, MZ937003.2; SARS-CoV-2, NC_045512.2; SARS-CoV-1, NC_004718.3; Rs4084, KY417144.1; RsSHC014, KC881005.1; WIV1, KF367457.1; Rs7327, KY417151.1; Rs4231, KY417146.1; LYRa11, KF569996.1; Pangolin GD-1, EPI_ISL_410721; Pangolin GX-P2V, EPI_ISL_410542; RhGB01, MW719567.1; Khosta-2, MZ190138.1; BtKY72, KY352407.1. The NBN Atlas datasets used for species niche modelling are listed in Supplementary Data 5.

Field-specific reporting

Please select the one below that is the best fit for your research. If you are not sure, read the appropriate sections before making your selection.

☒ Life sciences ☐ Behavioural & social sciences ☐ Ecological, evolutionary & environmental sciences

For a reference copy of the document with all sections, see nature.com/documents/nr-reporting-summary-flat.pdf

Life sciences study design

All studies must disclose on these points even when the disclosure is negative.

Sample size	No sample size calculations were performed. However, all pseudovirus assays throughout were repeated on the day with at least 3 replicates, which has proven in the past to be sufficient to see a significant effect. Assays were further performed on at least 3 separate occasions (details provided in figure legends). For the BLI experiments, sample size was not determined and assay results do not depend on sample size. Additionally, BLI measurements were repeated with multiple concentrations done by serial dilution were as per standard practice.
Data exclusions	No data was excluded from the analyses.
Replication	For pseudovirus assays, experimental data shown is representative of multiple experiments which replicated the findings. All assays throughout were repeated on the day with at least 3 replicates. Assays were further performed on at least 3 separate occasions (details provided in figure legends).<br><br>BLI measurements of SARS-CoV-2 and RhGB07 spikes with (b) hACE2 were done in two and three independent experiments, and at three and seven protein concentrations, respectively. BLI measurements for RhGB07 spike with R. ferrumequinum or M. lucifugus ACE2 were both repeated only once, but at seven protein concentrations.
Randomization	Randomization was not relevant for this study as assays were read in an unbiased manner - ie there was little or no interpretation involved that could be influenced by unconscious bias.
Blinding	Blinding was not possible/relevant for this study as assays were read in an unbiased manner - ie there was little or no interpretation involved that could be influenced by unconscious bias.

Reporting for specific materials, systems and methods

We require information from authors about some types of materials, experimental systems and methods used in many studies. Here, indicate whether each material, system or method listed is relevant to your study. If you are not sure if a list item applies to your research, read the appropriate section before selecting a response.

Materials & experimental systems

n/a	Involved in the study
☐	☒ Antibodies
☐	☒ Eukaryotic cell lines
☒	☐ Palaeontology and archaeology
☒	☐ Animals and other organisms
☒	☐ Human research participants
☒	☐ Clinical data
☒	☐ Dual use research of concern

Methods

n/a	Involved in the study
☒	☐ ChIP-seq
☒	☐ Flow cytometry
☒	☐ MRI-based neuroimaging

Antibodies

Antibodies used

mouse anti-tubulin (diluted 1/5,000; abcam; ab7291)
mouse anti-p24 (diluted 1/2,000; abcam; ab9071)
rabbit anti-SARS spike protein (diluted 1/2,000; NOVUS; NB100-56578)
rabbit anti-HA tag (diluted 1/2000; abcam; ab9110)
rabbit anti-ACE2 antibody (diluted 1/500; abcam; ab15348)
rabbit anti-Myc tag (diluted 1/2000; abcam; ab9106)
IRDye® 680RD Goat anti-mouse (diluted 1/10,000; abcam; ab216776)
IRDye® 680RD Goat anti-rabbit (diluted 1/10,000; abcam; ab216777)

Validation

These are all commercially available antibodies with validation data available on the manufacturer's website. All antibodies used were specifically against the human version of antigens and used in this manuscript against the human version - therefore species validation is irrelevant. All antibodies were specifically chosen as they had been validated by Western Blot by the manufacturers.

Eukaryotic cell lines

Policy information about cell lines

Cell line source(s)

HEK 293T - ATCC® CRL-11268;
Caco-2 - ATCC® HTB-37;
Calu-3 - ATCC® HTB-55;
NCTC clone 1469 ATCC® CCL-9.1

Authentication

We did not authenticate the cell lines.

Mycoplasma contamination

We did not test for mycoplasma.

Commonly misidentified lines
(See ICLAC register)

No commonly misidentified cell lines were used in the study.
